# The hidden universal distribution of amino acid biosynthetic networks: a genomic perspective on their origins and evolution

**DOI:** 10.1186/gb-2008-9-6-r95

**Published:** 2008-06-09

**Authors:** Georgina Hernández-Montes, J Javier Díaz-Mejía, Ernesto Pérez-Rueda, Lorenzo Segovia

**Affiliations:** 1 Departamento de Ingeniería Celular y Biocatálisis, Instituto de Biotecnología, Universidad Nacional Autónoma de México. Av. Universidad, Col. Chamilpa, Cuernavaca, Morelos, México, CP 62210; 2 Department of Biology, Wilfrid Laurier University, University Av. Waterloo, ON N2L 3C5, Canada; and Donnelly Centre for Cellular and Biomolecular Research, University of Toronto. College St., Toronto, ON M5S 3E1, Canada

## Abstract

**Background:**

Twenty amino acids comprise the universal building blocks of proteins. However, their biosynthetic routes do not appear to be universal from an *Escherichia coli*-centric perspective. Nevertheless, it is necessary to understand their origin and evolution in a global context, that is, to include more 'model' species and alternative routes in order to do so. We use a comparative genomics approach to assess the origins and evolution of alternative amino acid biosynthetic network branches.

**Results:**

By tracking the taxonomic distribution of amino acid biosynthetic enzymes, we predicted a core of widely distributed network branches biosynthesizing at least 16 out of the 20 standard amino acids, suggesting that this core occurred in ancient cells, before the separation of the three cellular domains of life. Additionally, we detail the distribution of two types of alternative branches to this core: analogs, enzymes that catalyze the same reaction (using the same metabolites) and belong to different superfamilies; and 'alternologs', herein defined as branches that, proceeding via different metabolites, converge to the same end product. We suggest that the origin of alternative branches is closely related to different environmental metabolite sources and life-styles among species.

**Conclusion:**

The multi-organismal seed strategy employed in this work improves the precision of dating and determining evolutionary relationships among amino acid biosynthetic branches. This strategy could be extended to diverse metabolic routes and even other biological processes. Additionally, we introduce the concept of 'alternolog', which not only plays an important role in the relationships between structure and function in biological networks, but also, as shown here, has strong implications for their evolution, almost equal to paralogy and analogy.

## Abstract 2

A core of widely distributed network branches biosynthesizing at least 16 out of the 20 standard amino acids is predicted using comparative genomics.

## Background

Metabolism represents an intricate set of enzyme-catalyzed reactions synthesizing and degrading compounds within cells. It is likely that a small number of enzymes with broad specificity existed in early stages of metabolic evolution. Genes encoding these enzymes probably have been duplicated, generating paralog enzymes that, through sequence divergence, became more specialized, giving rise, for instance, to the isomerases HisA (EC:5.3.1.16) and TrpC (EC:5.3.1.24), which act in histidine and tryptophan biosynthesis, respectively [1-4]. Additionally, gene duplication can promote innovations, generating enzymes catalyzing functionally different reactions, such as HisA, HisF (EC:2.4.2.-) and TrpA (EC:4.2.1.10). The classic view of metabolism is that relatively isolated sets of reactions or pathways are enough for the synthesis and degradation of compounds. The new perspective views metabolic components (substrates, products, cofactors, and enzymes) as nodes forming branches within a single network [5,6].

In the past few years, an increasing amount of information on metabolic networks from different species has become available [7-10], allowing for comparative genomic-scale studies on the evolution of both specific pathways [11,12] and whole metabolic networks [13-16]. Collectively, these studies highlight the contribution of gene duplication in the evolution of metabolism. Nevertheless, analog enzymes - those catalyzing the same reaction, even belonging to different evolutionary families - have been suggested to play an important role on this process as well [17]. This results, for instance, in three different types of acetolactate synthases (EC:2.2.1.6) acting in the biosynthesis of L-valine and L-leucine in *Escherichia coli*. Additionally, the modern perspective of metabolic processes has shown that evolutionary studies must include not only phylogenetic relationships among enzymes, but also the influence of some topological properties of metabolic networks [5,6,18-20]. One of these properties is the capability of metabolism to circumvent failures - for example, mutations promoting unbalanced fluxes - using alternative network branches and enzymes. Here, we introduce the term 'alternolog' to refer to these alternative branches and enzymes that, proceeding via different metabolites, converge in a common product. Some authors have suggested that alternative branches can contribute to genetic buffering in eukaryotes to a degree similar to gene duplication [18], but the role of these alternologs in the evolution of metabolism in other phylogenetic groups remains to be solved. In evolutionary terms, one can assume that the universal occurrence of some pathways and branches in modern species suggests that they existed in the last common ancestor (LCA). The evolution of these pathways and the emergence of paralogs, analogs and alternologs reflect an increased metabolic diversity as a consequence of increasing genome size, protein structural complexity and selective pressures in changing environments. In the evolution of amino acid biosynthesis, for instance, alternative pathways synthesizing L-lysine via either L,L-diaminopimelate or alpha-aminoadipate have been suggested to have developed independently in diverse clades [21-23]. The evolution of these pathways is closely related to the biosynthesis of L-arginine and L-leucine [22-24] and even to the Krebs cycle [24], but the origin of all these pathways is still under discussion. Diverse studies [6,25,26] have suggested that amino acids could be among the earliest metabolic compounds. However, two main questions have emerged from these studies: from what did their biosynthetic networks originate and how did they evolve? And how did gene duplication (paralogs), functional convergence (analogs) and network structural alternatives (alternologs) contribute to these processes? The purpose of this work is to broach these questions, combining both a network perspective and a comparative genomics approach. For this purpose we consider that the architecture of proteins preserves structural information that can be used to identify their relative emergence during the evolution of metabolism. Specifically, we identified a set of enzymes and branches that originated closer to the existence of the LCA, delimiting a core of enzyme-driven reactions that putatively catalyzed the biosynthesis of at least 16 out of the 20 amino acids in early stages of evolution. Additionally, we determined the contributions of biochemical functional alternatives to this core (paralogs, analogs, and alternologs) during the evolution of amino acid biosynthesis in diverse species.

## Results and discussion

### Biological distribution of amino acid biosynthetic networks

**Figure 1:**
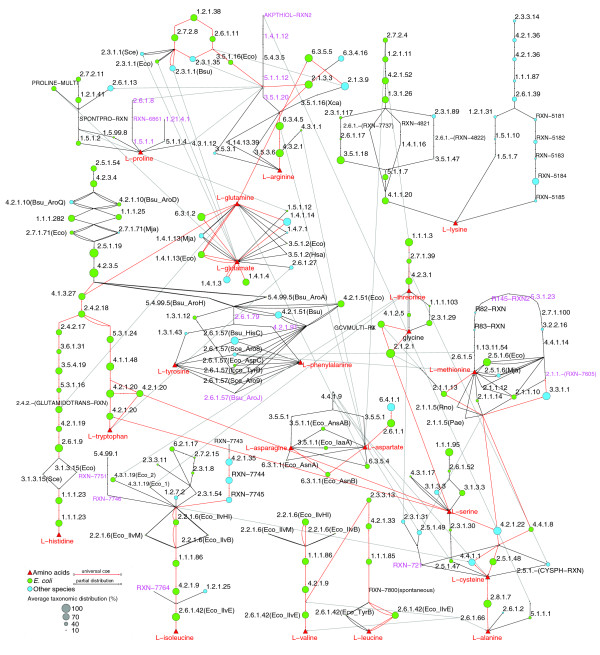
The amino acid biosynthetic network analyzed in this work. Bipartite amino acid biosynthetic network from multiple species. The 20 standard amino acids (red triangles) are shown as the ends of pathways. Green circles represent the canonical *E. coli *enzymes. Blue circles represent alternative enzymes (analogs and alternologs) from other species. The size of nodes corresponds to the normalized average taxonomic distribution of orthologs for each enzyme domain (domains in multimeric enzymes) catalyzing the corresponding reaction. The larger a node is the wider the distribution of orthologs for the corresponding enzyme across genomes. Red edges denote steps that could occur in the LCA based on the TDs of their catalyzing enzymes (Figures 2 and 4). Purple EC numbers correspond to reactions without known gene/enzymes. A detailed view of this network, including substrates and products, is provided in Additional data files 1 and 3, and the data for its construction are provided in Additional data files 2 and 4.

The origins and evolution of amino acid biosynthesis were assessed by analyzing the taxonomic distributions (TDs) of its catalyzing enzymes. Each enzyme's TD is a vector of ortholog distribution (presences/absences) in a set of genomes or clades (see Materials and methods). The rationale is that TDs provide clues concerning the relative appearance of enzymes, branches and pathways during the evolution of metabolism. We determined the TDs for 537 enzyme functional domains, catalyzing 188 reactions in the biosynthesis of amino acids from diverse species, in a set of 410 genomes (30 Archaea, 363 Bacteria and 17 Eukarya). To this end, we followed a two step strategy: first, we scanned the genomes to identify orthologs (best reciprocal hits (BRHs)) for the 113 amino acid biosynthetic enzymes from *E. coli *K12 defined in the EcoCyc database [8]: and second, a second set of ortholog, paralog, analog and alternolog enzymes and branches from different species, defined in the MetaCyc [9] and MjCyc [9] databases, was used to fill out the gaps in the *E. coli*-based TDs. Figure 1 shows a network formed by the 188 reactions analyzed in this work and the average distribution of orthologs for their catalyzing enzymes (see Materials and methods). We considered two broad categories for ortholog distribution: widely distributed enzymes, whose ortholog distribution is ≥ 50% across the clades analyzed here; and partially distributed enzymes, whose ortholog distribution is <50% across these clades. The wide distribution of enzymes, branches and pathways suggests their occurrence in the LCA, although these categories are simply a tool for presentation purposes. Even when a pathway shows a low average distribution of orthologs, some of its branches can be widely distributed across the three cellular domains (Archaea, Bacteria and Eukarya), and hence these branches might be present in the LCA. The opposite scenario can also take place, that is, some enzymes can exhibit a high average distribution, but they could be restricted to specific cellular domains or divisions, such as Bacteria or γ-proteobacteria, that are overrepresented in sequenced genomes. Thus, their distribution does not necessarily signify their occurrence in the LCA. For these reasons, we exhaustively examined the TDs of enzymes forming each branch within amino acid biosynthetic pathways. In the following sections we describe our main findings in decreasing order of average ortholog distribution, emphasizing the possible existence of some branches in the LCA.

### Nine amino acid biosynthetic pathways are widely distributed across the three domains of life, and eight of their branches probably occurred in the LCA

#### L-arginine

**Figure 2:**
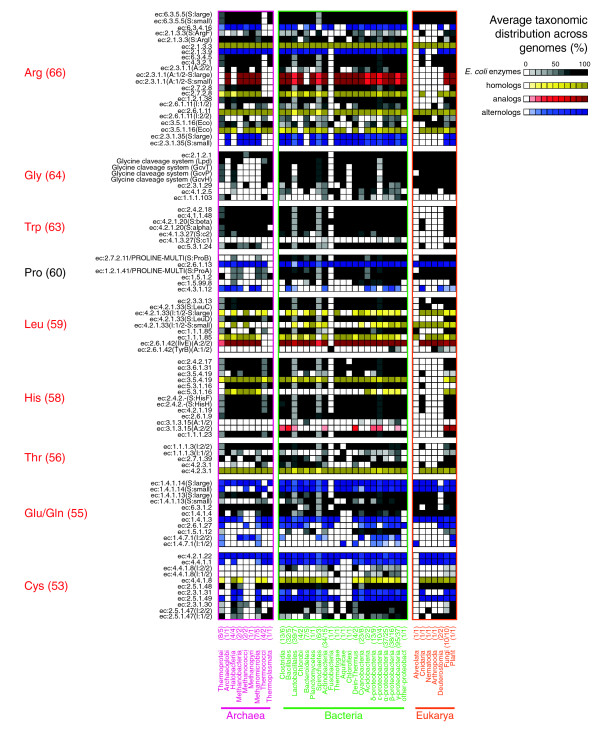
Average taxonomic distribution of amino acid biosynthetic enzymes widely distributed across the three domains of life. The TDs for enzymes catalyzing the amino acid biosynthetic pathways (vertical labels) were computed by searching for their ortholog distribution across diverse taxonomic groups (horizontal labels). The plot shows enzymes with an average normalized distribution ≥ 50% (see Materials and methods). Amino acid three letter codes in red denote amino acids whose biosynthesis probably occurred in the LCA (detailed in the main text). Four types of seeds were used to look for TDs: the canonical *E. coli *enzymes (gray scale); homolog enzymes - paralogs and orthologs - from other species showing a higher distribution than *E. coli *counterparts (yellow scale); analog enzymes - catalyzing the same reaction and coming from a different structural superfamily - (red scale); and alternolog enzymes and branches - converging in the same end compound, but proceeding via different metabolites - in other species (blue scale). In the vertical labels, subunits of multimeric enzymes are denoted with 'S', analog enzyme machinery is denoted with 'A' and isoenzymes are denoted with 'I'. For example, the annotation 'EC:3.5.1.1(Eco_Ans-AnsB)(A:1/2-I:1/2)' indicates that there are two analog EC:3.5.1.1 enzymes and this annotation corresponds to the first type (A:1/2). In turn, this type has two isoenzymes and this annotation corresponds to the first one (I:1/2), formed by AnsA and AnsB proteins in *E. coli*. The average distribution of orthologs for each route is shown in parentheses following amino acid three letter codes. Biosynthetic enzymes for each amino acid were sorted as they appear downstream in the metabolic flux.

**Figure 3:**
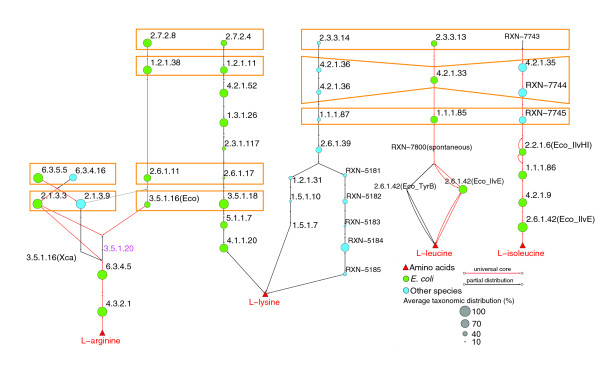
Retention of duplicates as groups instead of as single entities. Orange frames indicate pairs of duplicated genes (paralog enzymes) retained as groups instead of as single entities between the biosynthesis of L-arginine, L-lysine, L-leucine and L-isoleucine.

There are at least four L-arginine synthesis pathways, interplaying with the conversion of L-ornithine and citrulline, although they can be grouped in two superpathways (Figure 1). The first superpathway, involving carbamoyl-phosphate and N-acetyl-L-citrulline, can proceed via two alternolog branches: the first branch is the canonical *E. coli *pathway, catalyzed by two widely distributed enzymes, carbamoyl phosphate synthetase (EC:6.3.5.5) and ornithine carbamoyltransferase (EC:2.1.3.3). The second branch uses three enzymes (EC:6.3.4.16, EC:2.1.39 and EC:3.5.1.16), of which two are also widely distributed (Figure 2). Interestingly, EC:6.3.5.5 and EC:6.3.4.16 enzymes are paralogs, and EC:2.1.3.3 and EC: 2.1.39 are paralogs as well (Figure 3), representing an event of retention of duplicated genes as groups, instead of single entities. The retention of groups of duplicates has been suggested to play a significant role in the evolution of metabolism [16]. Alternatively, the second superpathway occurring via N-acetyl-L-ornithine is also widely distributed across the three domains, with the exception of animals, and shows three interesting TDs. First, using the *E. coli *enzymes as seeds for BRHs in this superpathway, we detected a small amount of orthologs in some clades, but using the ortholog sequences from *Saccharomyces cerevisiae*, *Methanocaldococcus jannaschii *and *Bacillus subtilis*, the gaps were filled in their respective phylogenetic groups (yellow squares in Figure 2), showing the importance of using enzymes from multiple species as queries instead of the simpler *E. coli*-centric strategies. Second, there are two analog N-acetylglutamate synthases (EC:2.3.1.1). The *E. coli*-type is a monomeric monofunctional enzyme, while the *B. subtilis*-type is a heterodimeric bifunctional enzyme (EC:2.3.1.1/2.3.1.35) whose constituents are proteolytically self-processed from a single precursor protein. Both types of enzymes are widely distributed across the three domains (Figure 2), although the *E. coli*-type was not identified in firmicutes, suggesting its displacement by the *B. subtilis*-type. Third, another retention of duplicated genes as groups, instead of as single entities, occurs between three consecutive steps in the biosynthesis of L-arginine/L-lysine [22]: EC:2.7.2.8/EC:2.7.2.4, EC:1.2.1.38/EC:1.2.1.11, EC:2.6.1.11/EC:2.6.1.17 and EC:3.5.1.16/EC:3.5.1.18 (Figure 3). In summary, we propose that not all pathways to synthesize L-arginine occurred in the LCA, only those proceeding via N-acetyl-L-ornithine and citrulline.

#### L-glycine

There are four branches to synthesize L-glycine. Two of them, involving the degradation of L-threonine (Figure 1), are partially distributed in Bacteria and Eukarya (Figure 2). In contrast, the other two branches, interconnected through 5,10-methylene-tetrahydrofolate, involve either the glycine-cleavage system or serine hydroxymethyltransferase (EC:2.1.2.1). Both branches are widely distributed across the three cellular domains (Figure 2). Indeed, EC:2.1.2.1 is one of the most widely distributed enzymes across all the species, probably as it also participates in folate biosynthesis, another broadly distributed pathway. Collectively, the distribution of these enzymes suggests that the LCA synthesized glycine via the branch of 5,10-methylene-tetrahydrofolate.

#### L-tryptophan

We found the five L-tryptophan biosynthetic enzymes widely distributed across the three domains of life, confirming previous reports [27]. Nevertheless, we did not identify orthologs for these enzymes in animals (Figure 2), with the exception of *Nematostella vectensis*, a cnidaria representative of early stages in animal evolution [28]. This indicates that some animals had a secondary loss of the L-tryptophan biosynthetic enzymes and also explains why this amino acid is essential for humans. Thus, the LCA probably was able to synthesize L-tryptophan in a similar fashion to contemporary species.

#### L-proline

There are at least six L-proline biosynthetic branches (Figure 1). Three of them converge in L-glutamate γ-semialdehyde and, judging from their TDs, ornithine-δ-aminotransferase (EC:2.6.1.13) is the most widely distributed enzyme within this pathway, even in some archaeal genomes (Figure 2). The other two branches have been biochemically characterized, although their catalyzing enzymes are unknown. The sixth branch, which directly converts L-ornithine to L-proline via ornithine cyclodeaminase (EC:4.3.1.12), was found in some Archaea and scarcely in Bacteria and Eukarya (Figure 2). Further analyses are necessary to corroborate experimentally the activities of these archaeal open reading frames, because the putative EC:2.6.1.13 enzymes do not have the canonical catalytic residues involved in this activity, and little information is known about the EC:4.3.1.12 activity. Thus, the archaeal biosynthesis of L-proline remains enigmatic and makes it difficult to infer if the LCA was capable of synthesizing L-proline.

#### L-leucine

The biosynthesis of L-leucine consists of five reactions following a mainly linear pathway (Figure 1). Using the *E. coli *and *M. jannaschii *sequences for BRHs, we detected that putative enzymes catalyzing the first three reactions are widely distributed (Figure 2). These three enzymes belong to a group of duplicated genes catalyzing consecutive steps in the biosynthesis of three amino acids, L-lysine, L-leucine and L-isoleucine (Figure 3). The evolutionary relationships between L-lysine and L-leucine biosynthesis have been documented previously [23,24,29]: we found that L-isoleucine biosynthesis is also implied in this phenomenon. These duplicates together with those from L-arginine/L-lysine biosynthesis support our previous report on the importance of the retention of duplicated genes as groups, instead of as single entities, in the evolution of metabolism [16]. The fourth reaction occurs spontaneously and does not require a catalyzing enzyme. Complementarily, the fifth step in *E. coli *is catalyzed by one out of the two analog branched-chain amino acid transferases (EC:2.6.1.42); one of them belongs to the D-amino acid aminotransferase-like PLP-dependent superfamily and is widely distributed across the three domains, including some animals. In contrast, the second EC:2.6.1.42 belongs to the PLP-dependent transferases superfamily and is sparsely distributed across genomes. Collectively, these observations suggest that the LCA was able to synthesize L-leucine-like contemporary species. Further biochemical characterization of animal open reading frames is necessary, as L-leucine is an essential amino acid for humans.

#### L-histidine

Structurally speaking, L-histidine and L-tryptophan biosynthesis are similar; both are mainly linear pathways diverging from anthranilate using EC:2.4.2.18 (Figure 1) and, given their wide distribution, they have been proposed to be ancient pathways. The L-histidine biosynthesis enzyme histidinol-phosphatase (EC:3.1.3.15) is the only enzyme from this pathway partially distributed across genomes (Figure 2). This is probably due to the existence of two analog EC:3.1.3.15 enzymes (*S. cerevisiae*- and *E. coli*-types). Both types are highly divergent in sequence, and when we relaxed the stringency of BRH analysis (increasing the threshold E-value from 10^-6 ^to 10^-1^), we detected orthologs in 84% and 40% of the analyzed genomes for the *S. cerevisiae *and *E. coli *types, respectively. The other enzymes analyzed in this study are not affected by the stringency of BRHs. Additionally, we found that animals, with the exception of *N. vectensis*, have experienced a secondary loss of the L-histidine biosynthetic machinery (Figure 2). Taking these results together, we suggest that the LCA had the same L-histidine synthesis pathway as extant species.

#### L-threonine

Two out of the three L-threonine biosynthetic enzymes from *E. coli *were found across the three domains. We did not find any orthologs in Archaea when we performed a genome scan with the *E. coli *threonine synthase (EC:4.2.3.1) as seed. Alternatively, when we used as seed an *M. jannaschii *paralog with the same function, we identified orthologs in Archaea (Figure 2). Again, this finding reinforces the importance of using enzymes from multiple species as seeds. Some animals apparently lost the biosynthetic machinery for this amino acid, but *N. vectensis *retained it. We suggest that the LCA could synthesize L-threonine like contemporary species.

#### L-glutamine and L-glutamate

As depicted in Figure 1, the inter-conversion of L-glutamine and L-glutamate can be performed by many alternolog enzymes. Both paralog glutamate synthases, the NADH dependent (EC:1.4.1.14) and the NADPH dependent (EC:1.4.1.13), produce L-glutamate from L-glutamine, and are widely distributed across the three domains (Figure 2). In the reverse direction, from L-glutamate to L-glutamine, we found that glutamine synthetase (EC:6.3.1.2), which is ATP dependent, is also widely distributed across the three domains. This suggests that the LCA was able to inter-convert L-glutamine and L-glutamate. But it leaves one open question: was the LCA capable of producing these amino acids independently of each other? Similarly to glutamate synthases, both paralog glutamate dehydrogenases, the NAD(P)^+^-dependent (EC:1.4.1.3) and the NADP^+^-dependent (EC:1.4.1.4) enzymes, produce L-glutamate from 2-oxoglutarate and ammonia, and are also widely distributed across the three domains. On the other hand, all other reactions synthesizing L-glutamine use L-glutamate as substrate and are sparsely distributed. In summary, we suggest that the LCA was able to synthesize L-glutamate from 2-oxoglutarate and inter-convert it with L-glutamine, but it is difficult to determine if the LCA was able to produce this last amino acid independently of the former one.

#### L-cysteine

There are at least four ways to synthesize L-cysteine (Figure 1). The most widely distributed, proceeding via cystathionine, uses cystathionine β-synthase (EC:4.2.1.22) and cystathionine γ-lyase (EC:4.4.1.1) and is documented as being eukaryotic-type, yet we found it distributed across the three domains (Figure 2). Alternatively, cystathionine-β-lyase (EC:4.4.1.8), cystathionine γ-synthase (EC:2.5.1.-) and O-succinylhomoserine(thiol)-lyase (EC:2.5.1.48) catalyze equivalent reactions and they are widely distributed in Bacteria and Eukarya. In contrast, an alternolog branch using EC:2.5.1.47 via O-acetyl-L-serine is sparsely distributed across genomes (Figure 2), while another branch without assigned enzymes (nor genes) uses O-acetyl-L-homoserine. These findings suggest that not all the L-cysteine biosynthetic pathways occurred in the LCA, but that the contemporary eukaryotic-like type could.

### Eight amino acid biosynthetic pathways are partially distributed across the three domains of life, and five of their branches probably occurred in the LCA

#### L-lysine

L-lysine biosynthesis has been used largely to exemplify the existence of alternolog branches in amino acid biosynthesis [21-23]. Six alternative pathways can be recognized for the biosynthesis of L-lysine (Figure 1), grouped in two superpathways proceeding via either L,L-diaminopimelate or alpha-aminoadipate. The superpathway involving L,L-diaminopimelate has four alternolog branches, corresponding to L-lysine biosynthesis types I, II, III and VI in MetaCyc; they share a common set of six reactions catalyzed by widely distributed enzymes. Four of these enzymes catalyze the upper steps of the superpathway, from aspartate kinase (EC:2.7.2.4) to dihydrodipicolinate reductase (EC:1.3.1.26), and form the pairs of duplicated genes between the biosynthesis of L-arginine/L-lysine (Figure 3). The other two enzymes (EC:5.1.17 and EC:4.1.120) catalyze the lower portion of the superpathway. The TDs of enzymes catalyzing intermediate steps in these alternologs are as follow. In the type I pathway (*E. coli*-type), which is catalyzed by three enzymes, only N-succinyl-L,L-diaminopimelate desuccinylase (EC:3.5.1.18) is widely distributed across the three domains. In the type II pathway (*B. subtilis*-type), catalyzed by the other three enzymes, only tetrahydrodipicolinate acetyltransferase (EC:2.3.1.89) is widely distributed in Bacteria, while it is absent in Archaea and Eukarya. The type III pathway of *Corynebacterium glutamicum *(EC:1.4.1.16) appears constrained to some actinobacteria and firmicutes, while the recently discovered type VI pathway, formed by a single enzyme, namely L,L-diaminopimelate aminotransferase (EC:2.6.1.-), seems to be specific for plants. These results illustrate a general finding of this work: linear pathways seem to be more widely distributed than bifurcating ones. As described above, L-histidine, L-tryptophan and L-leucine pathways support this observation, and correlate with previous studies showing that within amino acid biosynthesis, larger pathways tend to have lower rates of change in their structure than shorter pathways [31]. However, further studies on whole metabolic networks are necessary to assess the generality of this property in the evolution of metabolism. On the other hand, the second superpathway, proceeding via the degradation of alpha-aminoadipate, is formed by lineage specific type IV and V pathways that share a core of five reactions from homocitrate synthase (EC:2.3.3.14) to α-aminoadipate aminotransferase (EC:2.6.1.39). This core contains the four enzymes forming pairs of duplicated genes between the biosynthesis of L-leucine/L-lysine (Figure 3). The type V pathway, using N-2-acetyl-L-lysine (RXN-5181 to RXN-5185), was characterized in the Thermus-Deinocuccus lineage, and its representatives were found in Archaea and some Bacteria, while the type IV pathway, proceeding via saccharopine (EC:1.2.1.31 to EC:1.5.1.7), appears restricted to Eukarya and some Bacteria. Collectively, the TDs of these two superpathways show that alternative pathways have led the origin of the biosynthesis of L-lysine. None of these alternologs appears to be universally distributed and, thus, the LCA probably was not able to produce L-lysine using the set of enzymes analyzed here. Interestingly, both L-lysine biosynthetic superpathways retain groups of duplicated genes for the biosynthesis of L-leucine and L-arginine (Figure 3), which, as detailed above, probably occurred in the LCA. Thus, there is a possibility that L-lysine biosynthesis was incorporated into metabolism from L-leucine and L-arginine biosynthetic routes.

#### L-methionine

**Figure 4:**
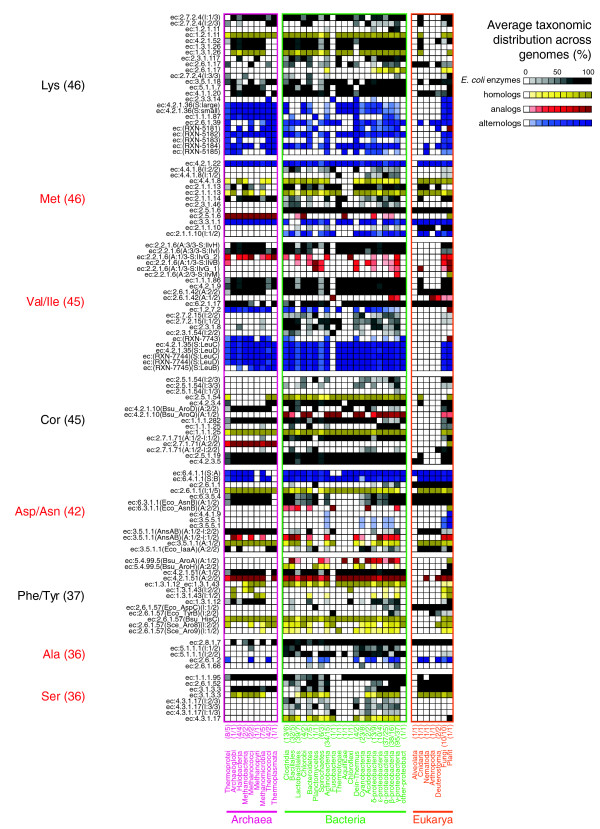
Average taxonomic distribution of amino acid biosynthetic enzymes partially distributed across the three domains of life. TDs for enzymes with an average normalized distribution <50% (see Materials and methods). Labels and colors are as in Figure 2.

The biosynthesis of L-methionine can be carried out by at least three different superpathways (Figure 1). One involves the degradation of cystathionine via homocysteine using either cystathionine β-synthase (EC:4.2.1.22) or cystathionine β-lyase (EC:4.4.1.8), followed by methionine synthase (EC:2.1.1.13). These three enzymes are widely distributed across the three domains (Figure 4) and, hence, this branch could occur in the LCA. Alternatively, the second superpathway, also called the L-methionine salvage cycle, which begins with EC:4.4.1.14 via S-adenosyl-L-methionine and finishes in L-methionine using EC:2.6.1.5 via 2-oxo-4-methylthiobutanoate (Figure 1), is widely distributed in Eukarya but almost absent in Archaea and Bacteria. An exception to this distribution is the step from L-methionine to S-adenosyl-L-methionine, which can be catalyzed by one of two analog methionine adenosyltransferases (EC:2.5.1.6). These analogs show an almost perfect anti-correlation in their TDs (Figure 4); one is restricted to Archaea, while the other occurs in Bacteria and Eukarya. Complementarily, a third superpathway, characterized in plants as the so-called S-adenosyl-L-methionine cycle, converts S-adenosyl-L-methionine to L-methionine via S-adenosyl-L-homocysteine (Figure 1). We found that one of this cycle's enzymes, S-adenosylhomocysteine hydrolase (EC:3.3.1.1), is widely distributed across the three domains. In summary, we suggest that the LCA was able to produce L-methionine, degrading cysthationine via homocysteine.

#### L-valine and L-isoleucine

The terminal four steps in the biosynthesis of L-valine and L-isoleucine employ a common set of widely distributed enzymes, from EC:2.2.1.6 to branched-chain amino-acid aminotransferase (EC:2.6.1.42) (Figure 4). This set was not found, however, in animals, again with the exception of *N. vectensis*. Complementarily, five alternolog branches can catalyze the initial steps of L-isoleucine biosynthesis, converging in 2-oxobutanoate, which is, in turn, a substrate of acetolactate synthase (EC:2.2.1.6) (Figure 1). We found that the canonical *E. coli *branch carrying out these steps via propionate uses EC:2.7.2.15 and EC:2.3.1.8 and is sparingly distributed among bacterial genomes. In contrast, the alternolog branch characterized in spirochaetes, proceeding via (R)-citramalate (Figure 1), uses isopropylmalate isomerase (EC:4.2.1.35) and β-isopropylmalate dehydrogenase (no EC number assigned), and both enzymes are widely distributed across the three domains (Figure 4). These results clearly exemplify that the *E. coli *canonical pathways are not necessarily the most widely distributed ones and, thus, alternolog pathways must be included in evolutionary analysis. Additionally, this branch participates in the retention of a group of duplicated genes catalyzing consecutive reactions in the biosynthesis of L-lysine, L-leucine and L-isoleucine (Figure 3). Taking together the wide distribution of the spirochaetes-like branch and the enzymes shared between L-valine and L-isoleucine biosynthesis, we suggest that the LCA and even contemporary species could combine these branches to synthesize both amino acids.

#### Chorismate

Chorismate is not an amino acid itself, but it is a key compound in the biosynthesis of aromatic amino acids and we consider the distribution of their catalyzing enzymes particularly interesting. The biosynthesis of chorismate comprises seven steps, the last two being catalyzed by two widely distributed enzymes, 3-phosphoshikimate-1-carboxyvinyltransferase (EC:2.5.1.9) and chorismate synthase (EC:4.2.3.5). Complementarily, the first two steps are catalyzed by enzymes widely distributed in Bacteria and some Eukarya, but absent in Archaea. A recent report suggesting a novel pathway for the biosynthesis of aromatic amino acids and *p*-aminobenzoic acid in the archaeon *Methanococcus maripaludis *helps to understand this distribution [32]. Additionally, three intermediate steps are catalyzed by scarcely distributed analog and alternolog enzymes as follows. First, the transformation of 3-dehydroquinate to 3-dehydro-shikimate can be catalyzed by two analog 3-dehydroquinate dehydratases (EC:4.2.1.10). *B. subtilis *possesses both analogs, while Archaea, some Eukarya and a few Bacteria carry only the type II enzyme (Figure 4) belonging to the aldolase (TIM-barrel) superfamily. In contrast, the majority of Bacteria, including *E. coli*, uses the type I enzyme (Figure 4) belonging to the 3-dehydroquinate dehydratase superfamily. Second, in *E. coli *there are two paralogs catalyzing the conversion of 3-dehydro-shikimate to shikimate. One of them, NADP^+^-dependent EC:1.1.1.25, is widely distributed, while EC:1.1.1.282 (using either NAD^+ ^or NADP^+^, and either quinate or shikimate) is sparsely distributed. In contrast, *B. subtilis *has only the NADP^+^-dependent shikimate dehydrogenase and, when its sequence is used as a seed for BRHs, we found more orthologs than with the *E. coli *counterparts (Figure 4). This finding is probably caused by cross-matches between the *E. coli *paralogs during the construction of TDs. Third, the transformation of shikimate to shikimate-3-phosphate can be catalyzed by two analog shikimate kinases (EC:2.7.1.71). The archaeal-type belongs to the GHMP kinase superfamily, while the bacterial/eukaryotic-type belongs to the superfamily of P-loop containing nucleoside triphosphate hydrolases. Interestingly, there is an almost perfect anti-correlation between the TDs of these enzymes (Figure 4). Animals, including *N. vectensis*, have lost all enzymes catalyzing intermediate steps in chorismate biosynthesis, supporting the fact that aromatic amino acids (L-histidine, L-trypthopan, L-phenylalanine, and L-tyrosine) are essential for humans. Summarizing, we found that the lower portion of chorismate biosynthesis, converting 3-dehydro-shikimate to chorismate, is widely distributed across the three domains, suggesting that it probably occurred in the LCA. In contrast, the upper and intermediate portions of this route appear to have originated independently in specific lineages during evolution.

#### L-aspartate and L-asparagine

The biosynthesis and inter-conversion of L-aspartate and L-asparagine are mediated by a diverse set of alternolog enzymes (Figure 1), most of which have been characterized in *E. coli *and are sparsely distributed. Nevertheless, aspartate aminotransferase (EC:2.6.1.1) and pyruvate carboxylase (EC:6.4.1.1) are able to produce L-aspartate from pyruvate, via oxaloacetate, and both enzymes are widely distributed across the three domains (Figure 4). Complementarily, the conversion of L-aspartate to L-asparagine can be carried out by three asparagine synthetases, two of which are glutamine dependent (EC:6.3.5.4) while the other is ammonia dependent (EC:6.3.1.1). Both EC:6.3.1.1 type 1 and EC:6.3.5.4 belong to the adenine nucleotide alpha hydrolases-like superfamily and are widely distributed across the three domains (Figure 4). In contrast, the production of L-aspartate and L-asparagine via 3-cyano-L-alanine, which is mediated by β-cyano-L-alanine-synthase (EC:4.4.1.9) and two paralog nitrilases (EC:3.5.5.1), appears to be restricted to plants, cyanobacteria and α-proteobacteria (Figure 4). This distribution could be the product of horizontal gene transfer among these clades, probably by symbiosis - as some α-proteobacteria are symbionts and parasites of plants - or by endosymbiosis - because cyanobacteria are considered descendants of plastid ancestors in plants. We did not detect any other possible horizontal gene transfer events in these routes using a database of putative horizontally transferred genes in prokaryotic complete genomes [33]. Finally, the two analog asparaginases (EC:3.5.1.1), converting L-asparagine to L-aspartate, show anti-correlated TDs. One of them, from the glutaminase/asparaginase superfamily, was found in Archaea, some Bacteria, Fungi and Animals (Figure 4), while the second one, from the superfamily of amino-terminal nucleophile aminohydrolases shows a distribution similar to that of EC:4.4.1.9 and EC:3.5.5.1. In summary, the LCA probably was not able to produce either L-aspartate or L-asparagine via the modern canonical alternologs (nitrilase and asparaginase), but could via the degradation of oxaloacetate using the branches described above.

#### L-tyrosine and L-phenylalanine

There are at least five branches diverging from prephenate for the biosynthesis of L-tyrosine and L-phenylalanine. Two of them proceed via phenylpyruvate and use one of the two widely distributed analog prephenate dehydratases (EC:4.2.1.51). Another two branches proceed via L-arogenate and use either arogenate dehydrogenase (EC:1.3.1.43) to synthesize L-tyrosine or arogenate dehydratase (EC:4.2.1.91) to synthesize L-phenylalanine. EC:1.3.1.43 occurs in Bacteria and some Archaea, while EC 4.2.1.91 has no assigned enzyme (nor gene) sequences. The fifth branch uses prephenate dehydrogenase (EC:1.3.1.12) followed by an aromatic-amino acid aminotransferase (EC:2.6.1.57). *E. coli*, *B. subtilis *and *S. cerevisiae *have two EC:2.6.1.57 and all of them can be classified in the PLP-dependent transferase superfamily, with the exception of AroJ in *B. subtilis*, whose sequence is unknown. However, it is difficult to establish orthology relationships between these enzymes because none of them are BRHs and, thus, are putatively paralogs. Apparently, this high diversity is maintained by differential expression and multifunctional properties of these enzymes. For instance, TyrB in *E. coli *is approximately 1,000-fold more active on aromatic substrates than its paralog AspC, which is more specific for aspartate. Similarly, in *B. subtilis*, HisC is more active than AroJ on phenylalanine and tyrosine, in spite of its primary activity on histidinol-phosphate, and *S. cerevisiae *uses Aro8 preferentially in anabolism and Aro9 in catabolism [34,35]. HisC could represent one of the most ancestral lineages in this family because it is the only member widely distributed across the three domains. This agrees with the fact that biosynthesis of L-histidine, the pathway in which HisC preferentially participates, is proposed to be ancestral (see above). The wide distribution of HisC and two analog EC:4.2.1.51 enzymes suggests that the biosynthesis of phenylalanine and tyrosine is also ancient. Nevertheless, the step preceding these enzymes, from chorismate to prephenate, can be catalyzed by one of the two analog chorismate mutases (EC:5.4.99.5); these show a sparse distribution, with some representatives in firmicutes, proteobacteria and plants. For instance, *E. coli *possesses two paralog EC:5.4.99.5 enzymes belonging to the chorismate mutase II superfamily and they are fused to domains catalyzing EC:1.3.1.12 and EC:4.2.1.51 activities, while *B. subtilis *also has two EC:5.4.99.5 enzymes, one from the chorismate mutase II superfamily and the other from the YjgF-like superfamily. Neither of these EC:5.4.99.5 domains is widely distributed; thus, it is difficult to establish whether the LCA was able to synthesize L-phenylalanine and L-tyrosine.

#### L-alanine

There are four alternolog single steps to synthesize L-alanine (Figure 1). One of them uses cysteine desulfurase (EC:2.8.1.7) to degrade L-cysteine and is widely distributed across the three domains. Given that L-cysteine biosynthesis probably occurred in the LCA (see above), we suggest that biosynthesis of L-alanine could occur in the LCA via this step. In contrast, alanine racemase (EC:5.1.1.1) isomerizes D-alanine to L-alanine and is constrained to Bacteria. Alanine aminotransferase (EC:2.6.1.2) converts L-glutamate and pyruvate to L-alanine and is widely distributed in Eukarya but poorly represented in Bacteria and Archaea, whereas valine-pyruvate aminotransferase (EC:2.6.1.66) degrades L-valine to L-alanine and was detected only in few Bacteria (Figure 4).

#### L-serine

Finally, there are four proficient branches to synthesize L-serine. The first, proceeding via 3-phospho-hydroxypyruvate, is catalyzed by three enzymes, two of them, 3-phosphoglycerate dehydrogenase (EC:1.1.1.95) and 3-phosphoserine phosphatase (EC:3.1.3.3) are widely distributed, but the third, phosphoserine aminotransferase (EC:2.6.1.52) is restricted to Eukarya and some Bacteria. The second branch is a single step converting ammonia and pyruvate to L-serine by L-serine ammonia-lyase (EC:4.3.1.17), and is restricted to some Bacteria. The third and fourth branches are closely related to the biosynthesis of L-cysteine and L-methionine, inter-converting cystathionine and homocysteine by either EC:4.2.1.22 or EC:4.4.1.8, which are widely distributed across the three domains (see above). Given that L-cysteine and L-methionine could exist in the LCA, the biosynthesis of L-serine could also exist via these enzymes. In fact, this chain of widely distributed enzymes can be extended to the biosynthesis of L-alanine (Figure 1), and all of them together constitute the larger succession of reactions that probably existed in the LCA.

**Figure 5:**
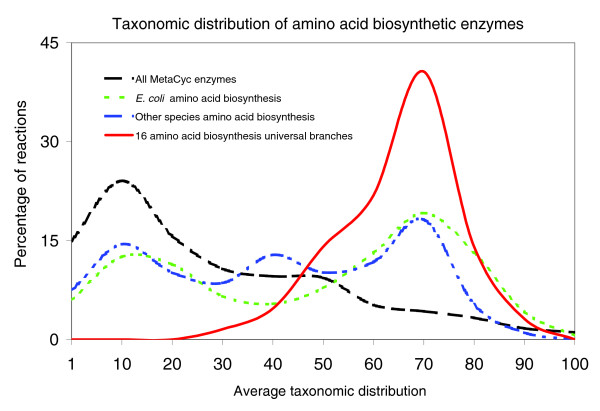
Taxonomic distribution of amino acid biosynthesis. Conservation of amino acid biosynthesis from the *E. coli*-centric and multi-organismal seed perspectives. The general trend in the whole of metabolism (MetaCyc), using a manually depurated set of enzyme domains, is also shown. The 16 amino acid biosynthesis universal branches show a maximum of around 45% of reactions (y-axis) in 70% of sampled genomes (x-axis) when all MetaCyc enzymes have a maximum of 24% of reactions in only 10% of genomes.

In summary, our results have uncovered a set of 64 enzyme domains participating in the biosynthesis of at least 16 out of the 20 proteinogenic amino acids that tentatively occurred in the LCA. Figure 5 shows a marked bias in the taxonomic distribution of this set of domains with respect to the general trend for the whole metabolism and other less conserved parts of amino acid biosynthesis, suggesting that branches in other metabolic processes could also posses a hidden universality.

## Conclusion

We have carried out a comprehensive analysis of the origin and evolution of amino acid biosynthesis. Our strategy combines genomic tools with a network perspective to identify a core of widely distributed enzymes that probably occurred in the LCA, synthesizing at least 16 out of the 20 standard amino acids. This proposal does not imply, however, that the full biosynthetic routes for these 16 amino acids appeared early, but only some of their branches that could satisfy the minimal biochemical and structural requirements. It is important to note that some species such as parasites and free living animals, including mammals, can lack significant portions of this 'universal' set because they can import amino acids from their hosts or include it in their diet. In parasites, these absences have been attributed to secondary loss. Our results show that most basal animal lineages and other Eukarya posses these universal branches and, thus, their absence in the animal kingdom apparently is also due to secondary losses. Further studies on the possible occurrence of the remaining four standard amino acid biosynthetic routes - for L-proline, L-lysine, L-phenylalanine and L-tyrosine - are necessary as some portions of these pathways are also widely distributed and some 'lost' reactions could fill the gaps.

One of the major biological roles of amino acids is that they are protein constituents; thus, an emerging question from our results is whether this core of amino acids could be sufficient for the LCA protein repertoire? Recently, Atchley *et al*. [36], grouped amino acids according to almost 500 attributes, ranging from structural to biochemical and biophysical properties, producing a multidimensional representation of amino acid variability. Mapping the putative core of 16 ancient amino acid biosynthetic branches onto the Atchley *et al*. plot (Figure 1 in Ref. [36]) suggests that the LCA was able to populate all the regions of amino acid variability space, so this core could be sufficient for protein functions in early biosystems. Most of the universal branches found in this work are connected to each other (Figure 1), allowing the possibility that they feedback and complete a minimal set of enzyme-driven reactions for the biosynthesis of amino acids. Interplay between the variability and selective pressures resulting from increasing genome sizes and protein structure complexity could promote the incorporation of novel amino acids to this core.

Additionally, we identified alternative branches and routes (paralogs, analogs and alternologs) reflecting the adoption of specific amino acid biosynthetic strategies by taxa, probably due to differences in their life-styles. Eleven out of the twenty amino acid biosynthetic routes revealed an important contribution of paralogy to the generation of diversity. In particular, we corroborated that the retention of gene duplicates as groups, instead of as single entities, is an important factor in the evolution of metabolism. Furthermore, analog enzymes contribute in eight out of the twenty standard amino acid biosynthetic routes, while alternolog routes participate in nine. This implies that analog enzymes and alternolog branches contribute almost as much as gene duplication to genetic buffering in the biosynthesis of amino acids. Further studies are necessary to determine the generality of these observations and to complement them with observations from alternative reactions modeling fluxes in metabolism [37]. In conclusion, we suggest that despite a core of amino acid biosynthetic branches being inherited from ancient systems, the whole contemporary repertoire has been originated independently by lineages according to their environmental resources as reflected by the high diversity of anabolic branches.

In this sense, we consider that one of the goals of the two step strategy presented here (*E. coli *and multi-organismal TD seeds) is that it uses not only a traditional model organism for genomic analyses, but also as many species as available in current databases. This is important because 8 out of the 20 amino acid biosynthetic routes (L-cysteine, L-serine, L-alanine, L-isoleucine, L-arginine, L-aspartate, L-proline and L-methionine) were quite sparingly distributed from an *E. coli*-centric perspective, but widely distributed when adding the orthologs, paralogs, analogs and alternologs from other species, revealing the universal nature of some of these routes. Further studies are necessary to determine the generality of these findings, not only in metabolic networks but also in other biological processes.

## Materials and methods

### Network reconstruction

In bipartite metabolic networks, there are two sets of nodes - enzymes and compounds (substrates, products and cofactors) - and edges relating enzymes with compounds occurring in the same reaction. For instance, if reaction R1 consumes compound C1 and produces C2 and C3, and it is catalyzed by enzyme E1, the following edges are established: C1 → E1, E1 → C2, and E1 → C3. In reversible reactions a second group of links from products to enzymes and, in turn, from enzymes to substrates is added. In this work we reconstructed the bipartite networks derived from three metabolic databases: EcoCyc v8.0 [8] for *E. coli*, MjCyc [10] for *M. jannaschii *and MetaCyc v8.0 [9] for multi-organismal assignments. To obtain information concerning the nodes and edges for each reaction, we used the following files from EcoCyc and MetaCyc: reactions.dat (substrate/product), enzrxns.dat (reversibility) and reaction-links.dat (EC numbers). From MjCyc the corresponding information was retrieved manually from the database's web page. Networks derived from these databases were merged and prepared for presentation with Cytoscape v2.5.2 [38]. Amino acids were highlighted (red triangles in Figure 1) to denote terminal points of pathways and branches into this network. For clarity in presentation, the most highly connected compounds (mainly cofactors) and the terminal non-amino acid metabolites were removed from the network. Additional data file 2 lists these compounds and contains the pairs of nodes used to construct Figure 1. Multifunctional enzyme sequences were split manually according to their functional domain assignments from Swiss-Prot [39]. Thus, in Figure 1 each node represents one reaction catalyzed by a functional domain (or domains in multimeric enzymes). Analogue enzymes - those catalyzing the same reaction but possessing different folds - were detected by comparing the structural domain content among proteins according to the Superfamily database v1. 69 [40] using HMMer [41]. Additional data file 2 contains details for the final set of 537 enzyme functional domains analyzed in this work as well as 32 reactions without known gene/enzymes. Alternologs were detected by manual inspection of the network in Figure 1, looking for branches that, proceeding via different metabolites, converge in a given compound, generally in an amino acid.

### Taxonomic distribution

Amino acid sequences from 537 enzymes (functional domains) were tracked in completely sequenced genomes using BLASTP (cutoff E-value = 10^-20^, and identity percentage >95), this was carried out to obtain the corresponding genomic sequences used as seeds for ortholog detection. Sixty-nine enzymes were excluded because they do not have assigned enzyme (gene) sequences (Additional data file 2). Each genomic sequence seed was used for ortholog detection across 410 non-obligate parasitic genomes (30 Archaea, 363 Bacteria and 17 Eukarya) representing 297 species from 192 genera (Additional data file 2), following the BRH criterion using BLASTP with a cut E-value of 10^-5^, and a minimum alignment coverage for query and/or subject sequence ≥ 50%. Genomes with less than 1,500 predicted open reading frames (mainly from obligate parasitic genomes) were eliminated from this analysis because they have experienced extensive secondary loses of anabolic enzymes in their genomes, which could introduce noise in TDs. Sequences in the same genome with >95% identity estimated with CD-HIT [42] were grouped into clusters. As reported [43], this procedure reduces the frequency of false-negative results caused by cross-matches between highly similar sequences within a genome. Given the redundancy of sequenced strains for some bacterial species, we systematically depurated the original set of genomes, attempting to obtain a normalized measure of ortholog distribution according to the following steps. In step 1, a TD of enzymes versus species was constructed assigning a value of1 to enzymes with orthologs (BRHs) in ≥ 50% of strains from each species. Otherwise a value of 0 was assigned to the corresponding bit. In step 2, a TD of enzymes versus genera was constructed. In this TD, each vector represents the percentage of species having a value of 1 (assigned in step 1) for each genus. In step 3, a TD of enzymes versus clades was constructed. In this TD each vector represents an average of the genera percentages obtained in step 2, for each clade. Clades correspond to the taxonomic categories from the Kyoto Encyclopedia of Genes and Genomes (KEGG) [7]. This procedure provides a 'normalized average distribution' of enzymes across genomes. The final set of strains and genera for each clade is shown in Additional data file 2.

## Abbreviations

BRH, best reciprocal hits; LCA, last common ancestor; TD, taxonomic distribution.

## Authors' contributions

GHM and JJDM carried out the analyses under the supervision of EPR and LS. All the authors planned the project and wrote the manuscript.

## Additional data files

The following additional data are available with the online version of this paper. Additional data file 1 is a graph showing a detailed view of the bipartite network analyzed in this work. Additional data file 2 provides details of enzymes analyzed in this work. Additional data file 3 is a detailed view of pathways and branches analyzed in this work. Additional data file 4 is a bipartite graph of the final metabolic network analyzed in this work (after hub and end compounds removal).

## Supplementary Material

Additional data file 1 As a complement to Figure 1, this file contains a detailed graph of the amino acid biosynthetic network analyzed in this work, including substrates and products. Additional data file 4 contains the network in xgmml format. Click here for file

Additional data file 2 Details on the 537 enzyme functional domains catalyzing 188 reactions in the biosynthesis of amino acids in diverse species according to EcoCyc, MetaCyc and MjCyc. The following details are provided: participating pathway(s); code for participating reactions in EcoCyc, MjCyc and MetaCyc; EC number(s); isoenzymes, multimeric components and analog enzymes; original species bearing the enzyme; genome carrying the enzyme; GI of genomic sequence; amino acid sequence - split in multifunctional enzymes; structural domains; average normalized distribution of orthologs across genomes; phylogenetic profile. Additionally, tables on the genomes included in this study, the connectivity of compounds and the node pairs forming the network in Figure 1 are provided. Click here for file

Additional data file 3 Detailed view from Figure 1 for pathways in the following order: **(a) **Arg, **(b) **Gly-Thr, **(c) **Trp, **(d) **Pro, **(e) **Ile-Val-Leu, **(f) **His, **(g) **Glu-Gln, **(h) **Ala-Cys-Ser-Met, **(i) **Lys, **(j) **Cor, **(k) **Asp/Asn, **(l) **Phe/Tyr. Nomenclature is as in Figure 1. Click here for file

Additional data file 4 Bipartite graph of the final metabolic network analyzed in this work (after hub and end compounds removal). Click here for file
