# Supplementary figures and images for: The hidden universal distribution of amino acid biosynthetic networks: a genomic perspective on their origins and evolution
Source: Genome Biol. 2008 Jun 9;9(6):R95. doi: 10.1186/gb-2008-9-6-r95 (PMC2481427; doi:10.1186/gb-2008-9-6-r95)

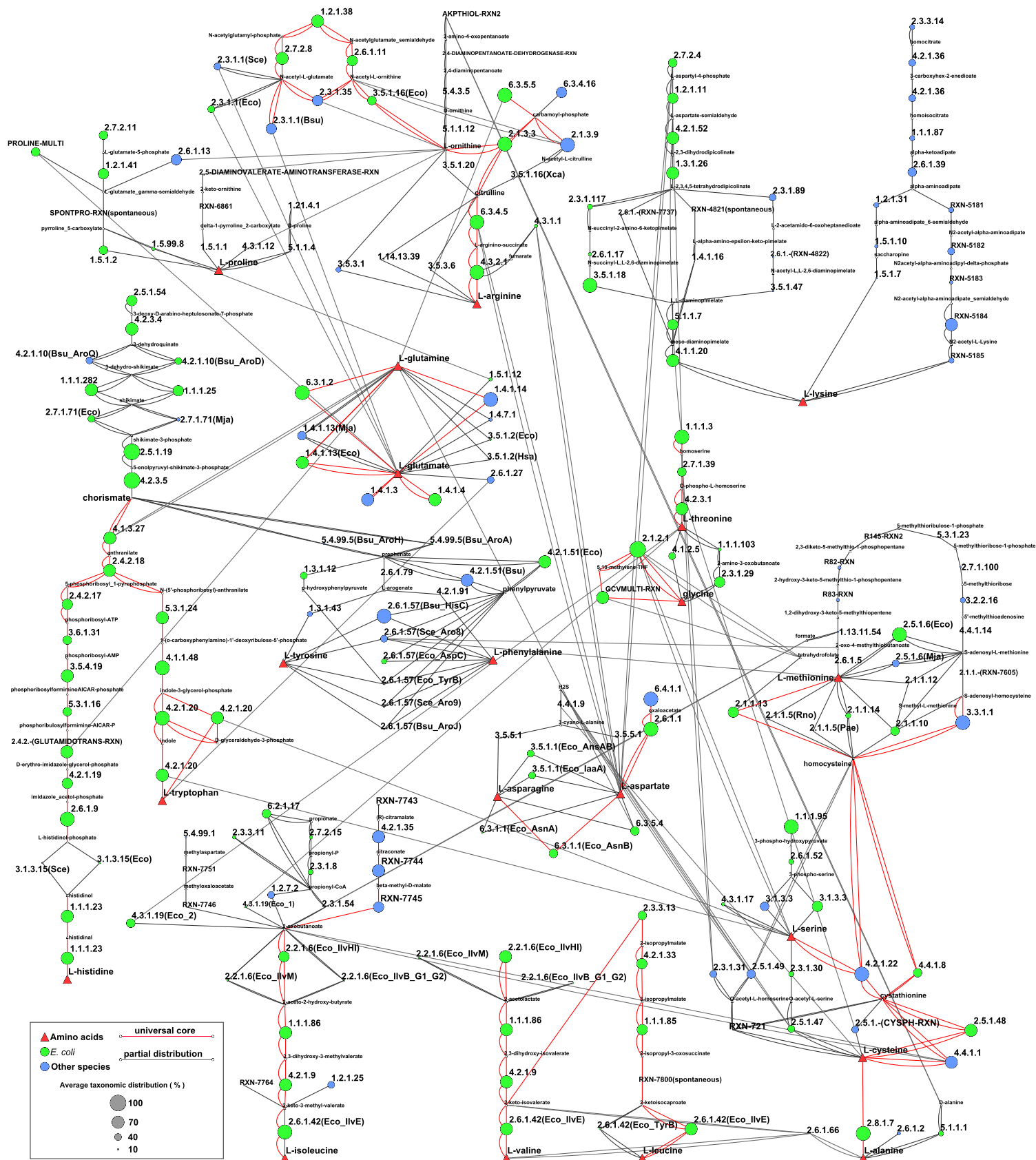

Supplement: Additional data file 1 — As a complement to Figure 1, this file contains a detailed graph of the amino acid biosynthetic network analyzed in this work, including substrates and products. Additional data file 4 contains the network in xgmml format. [file gb-2008-9-6-r95-S1.pdf]

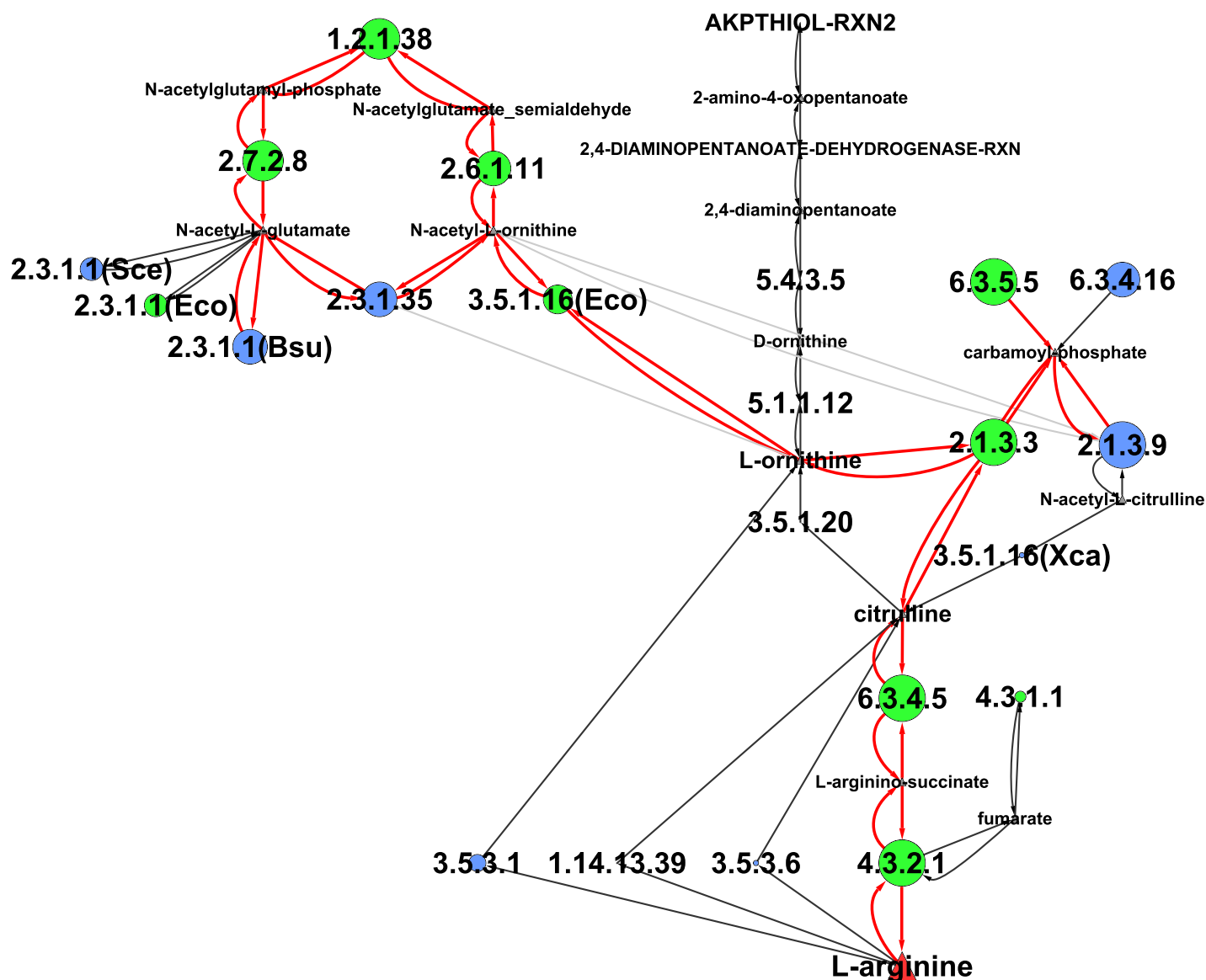

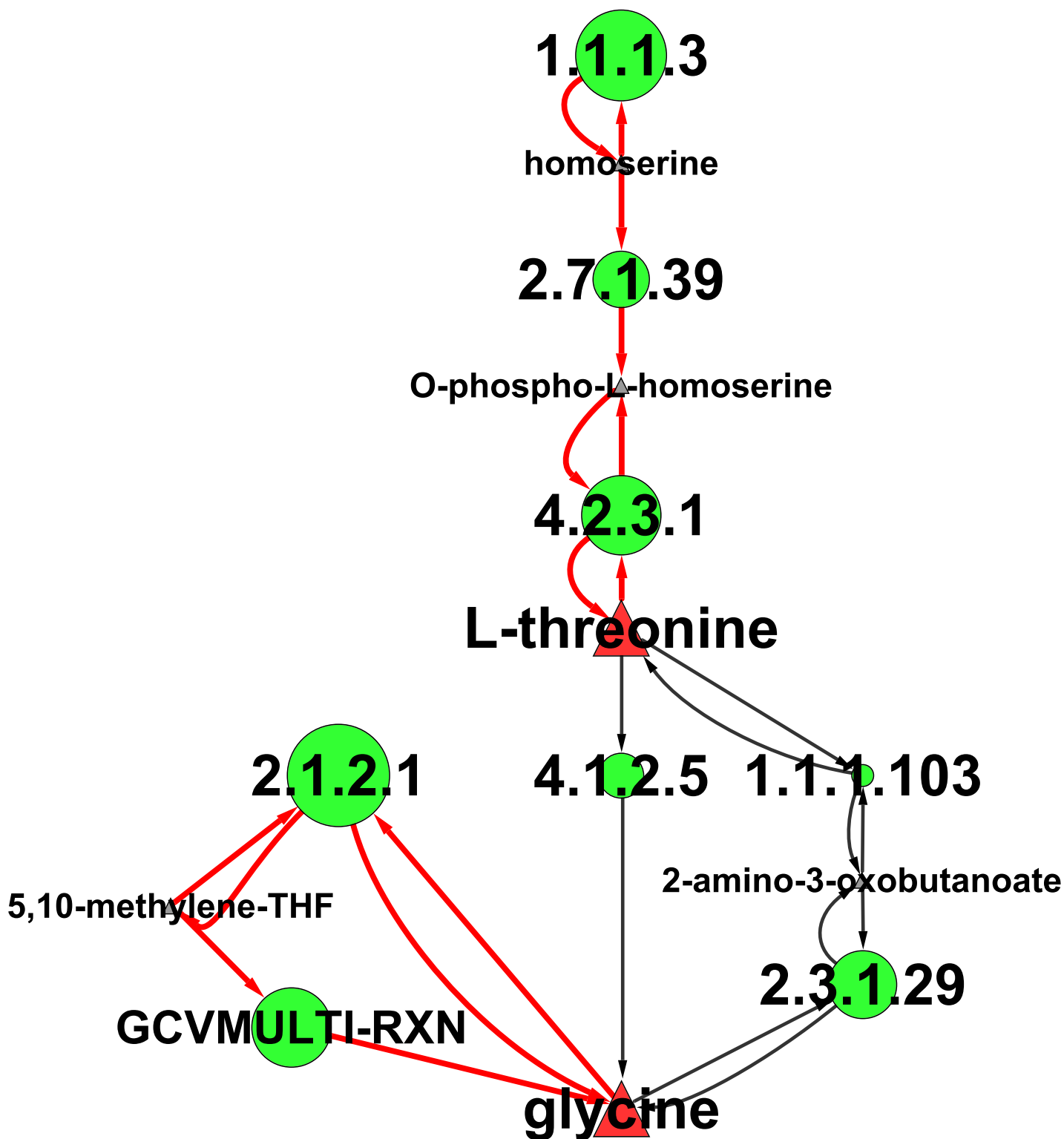

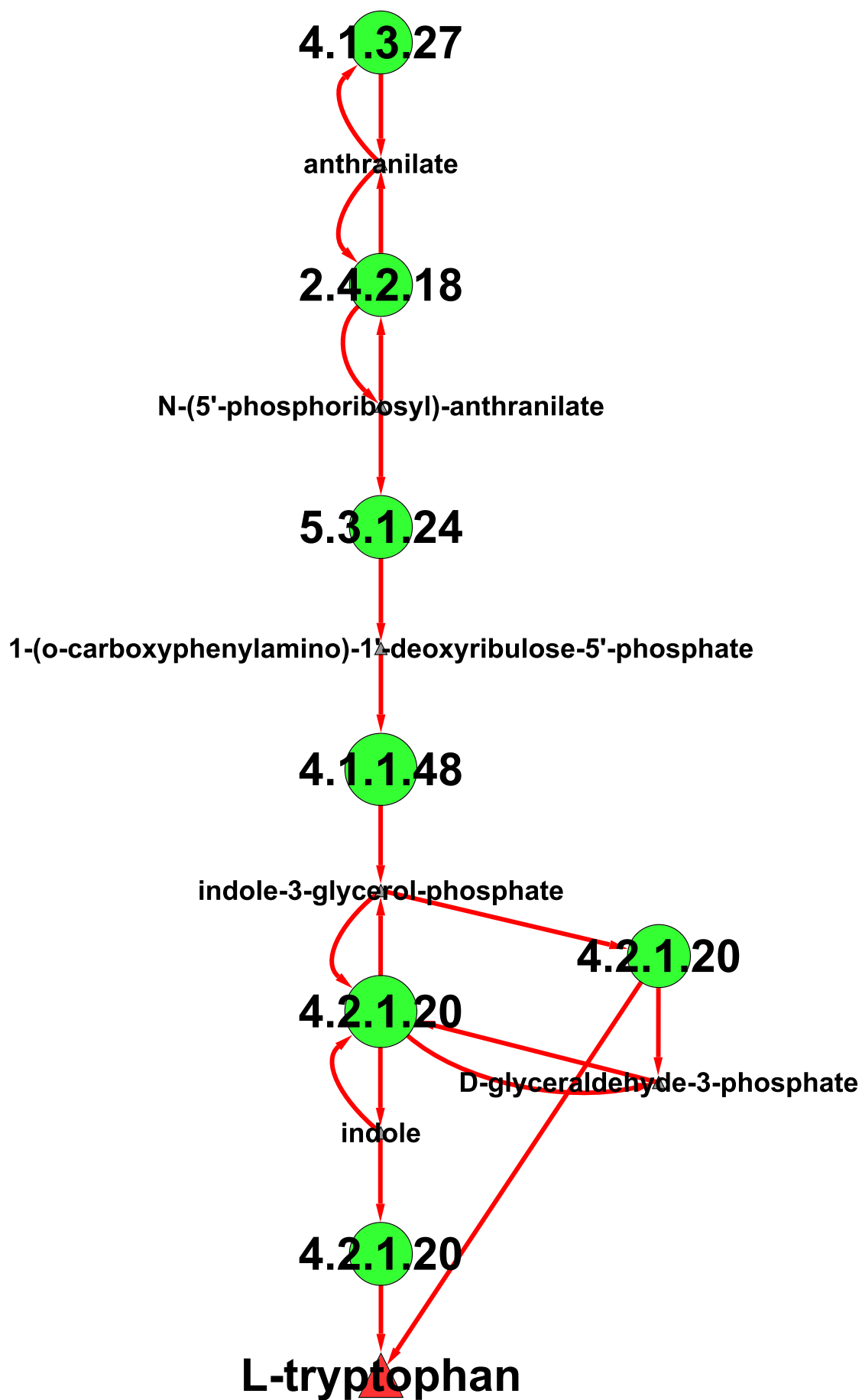

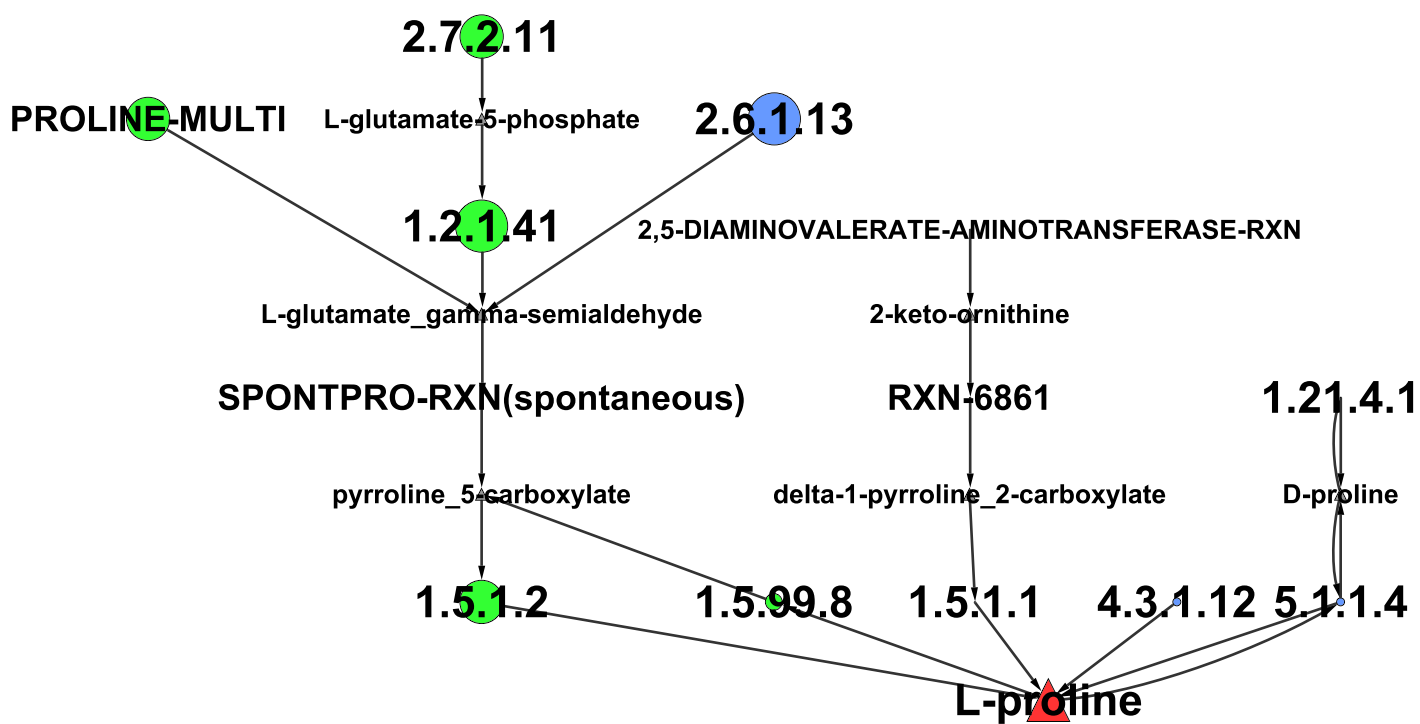

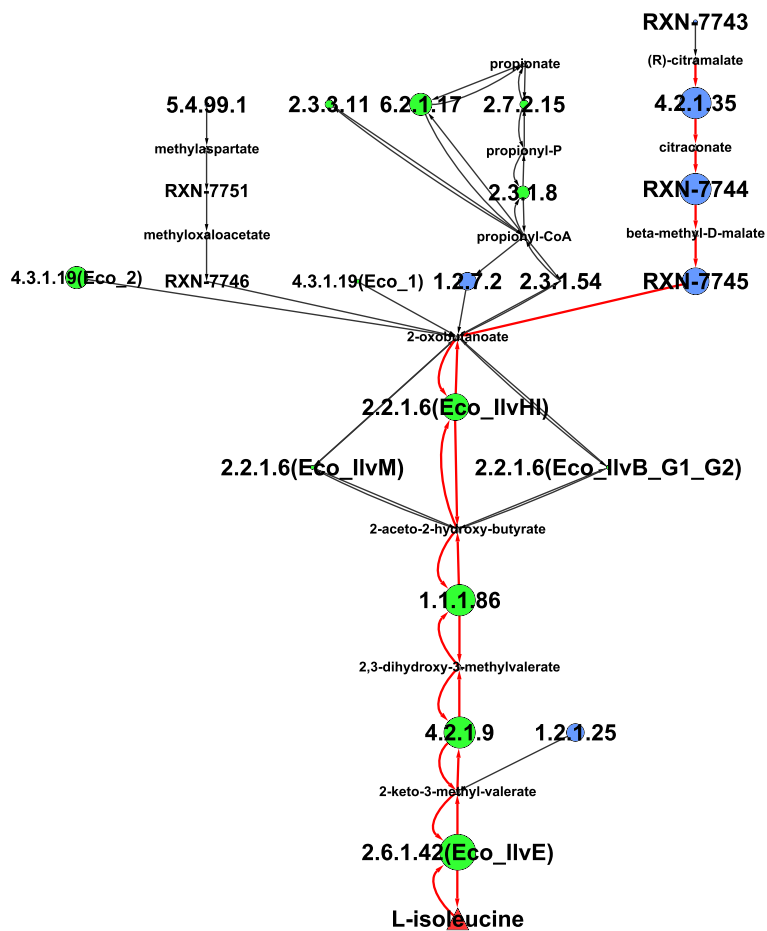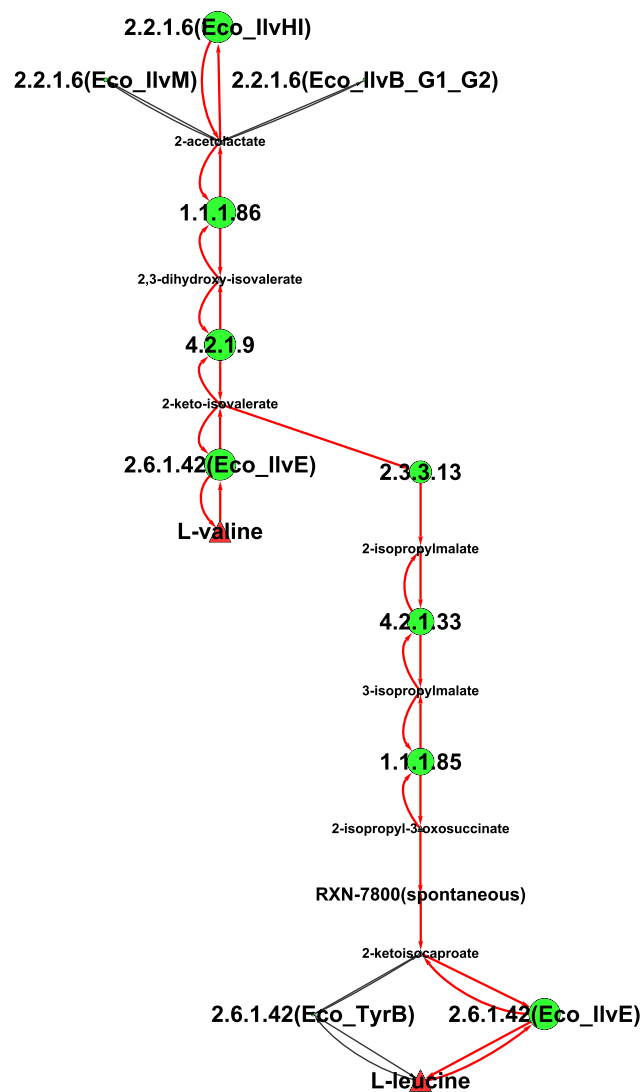

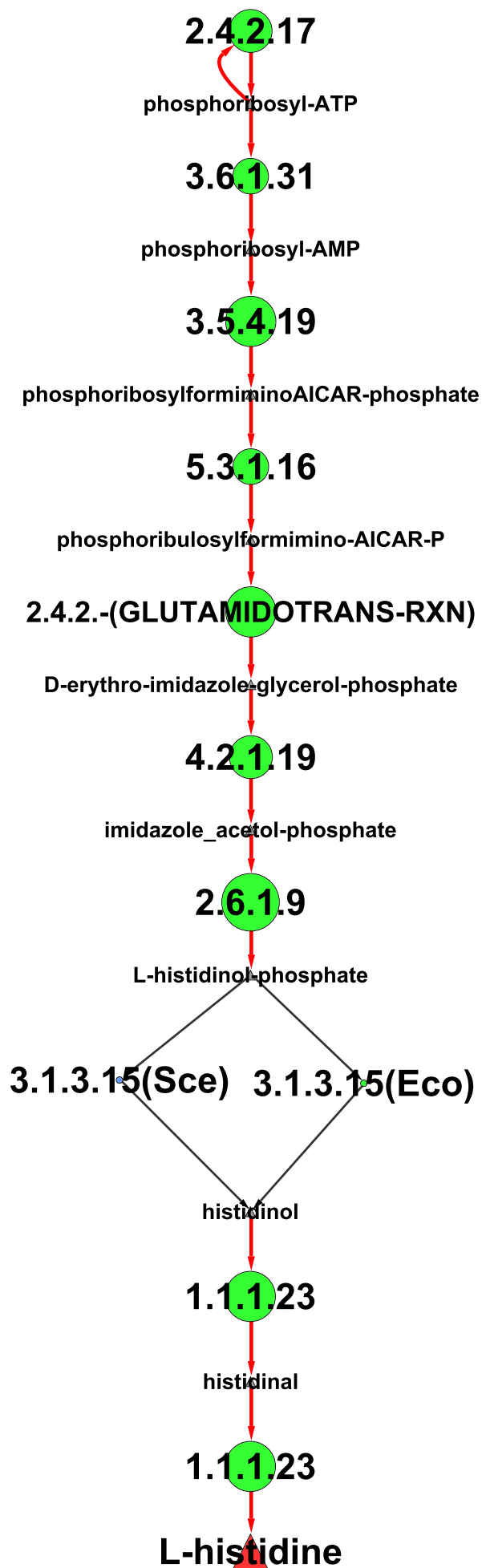

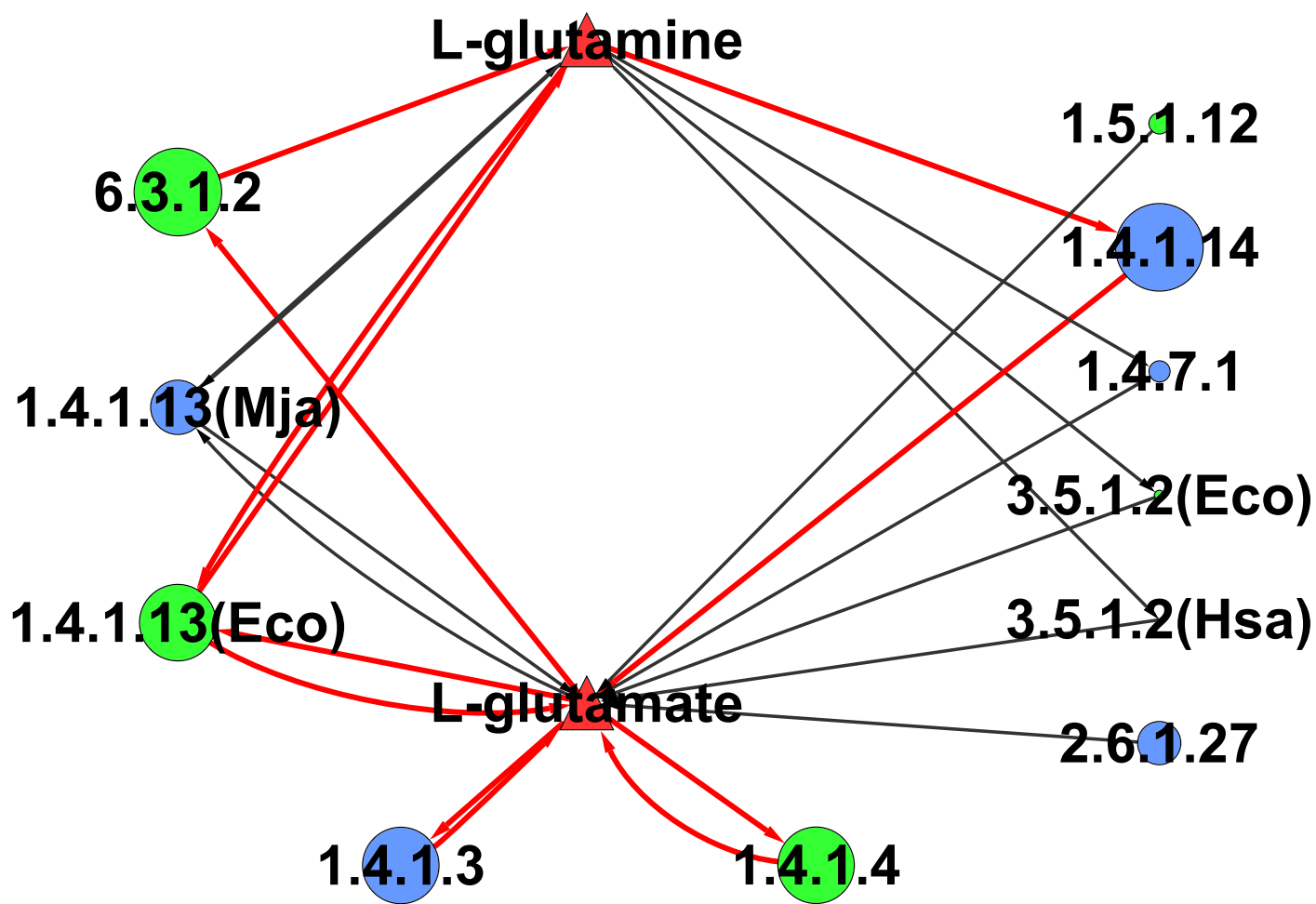

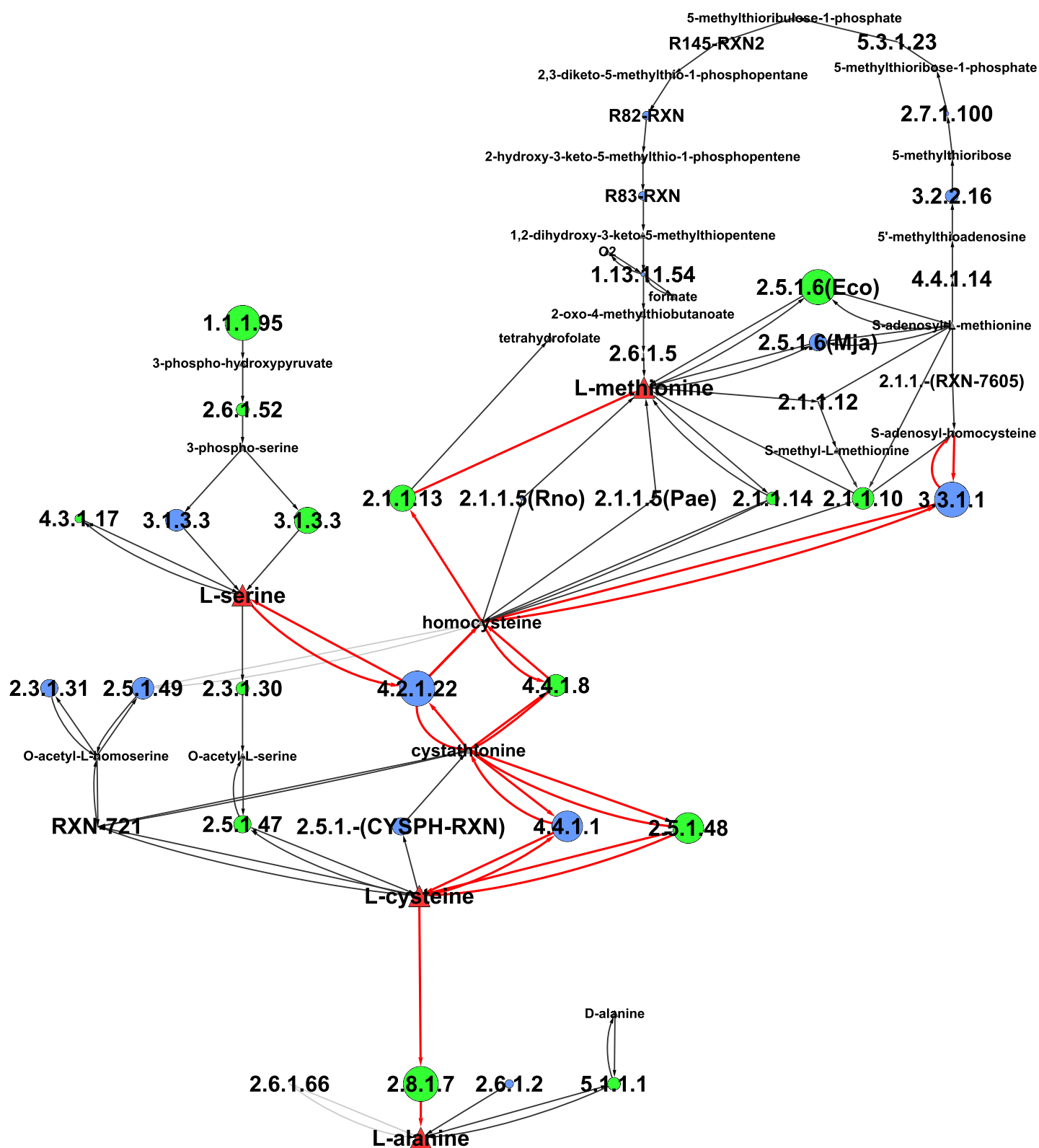

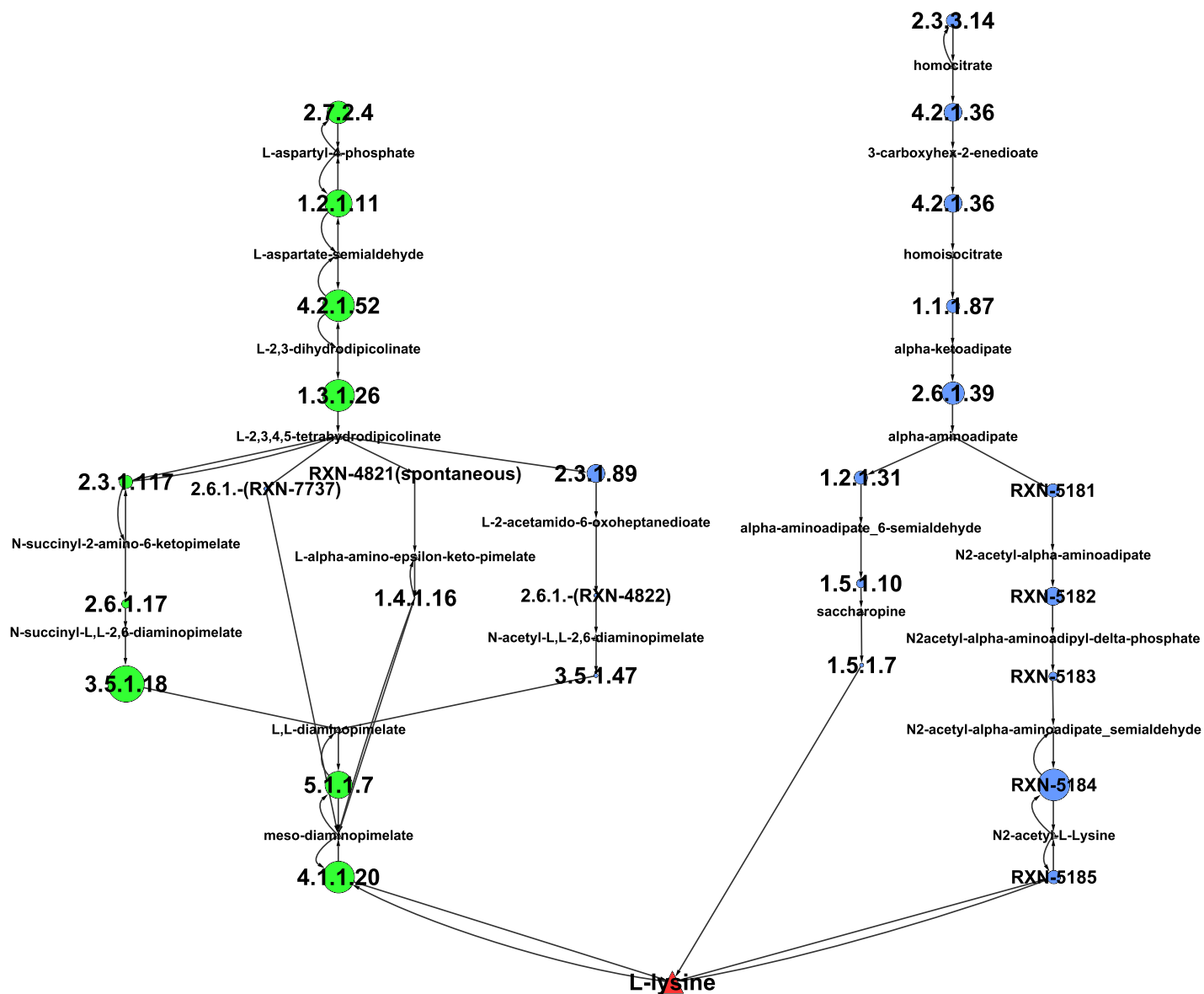

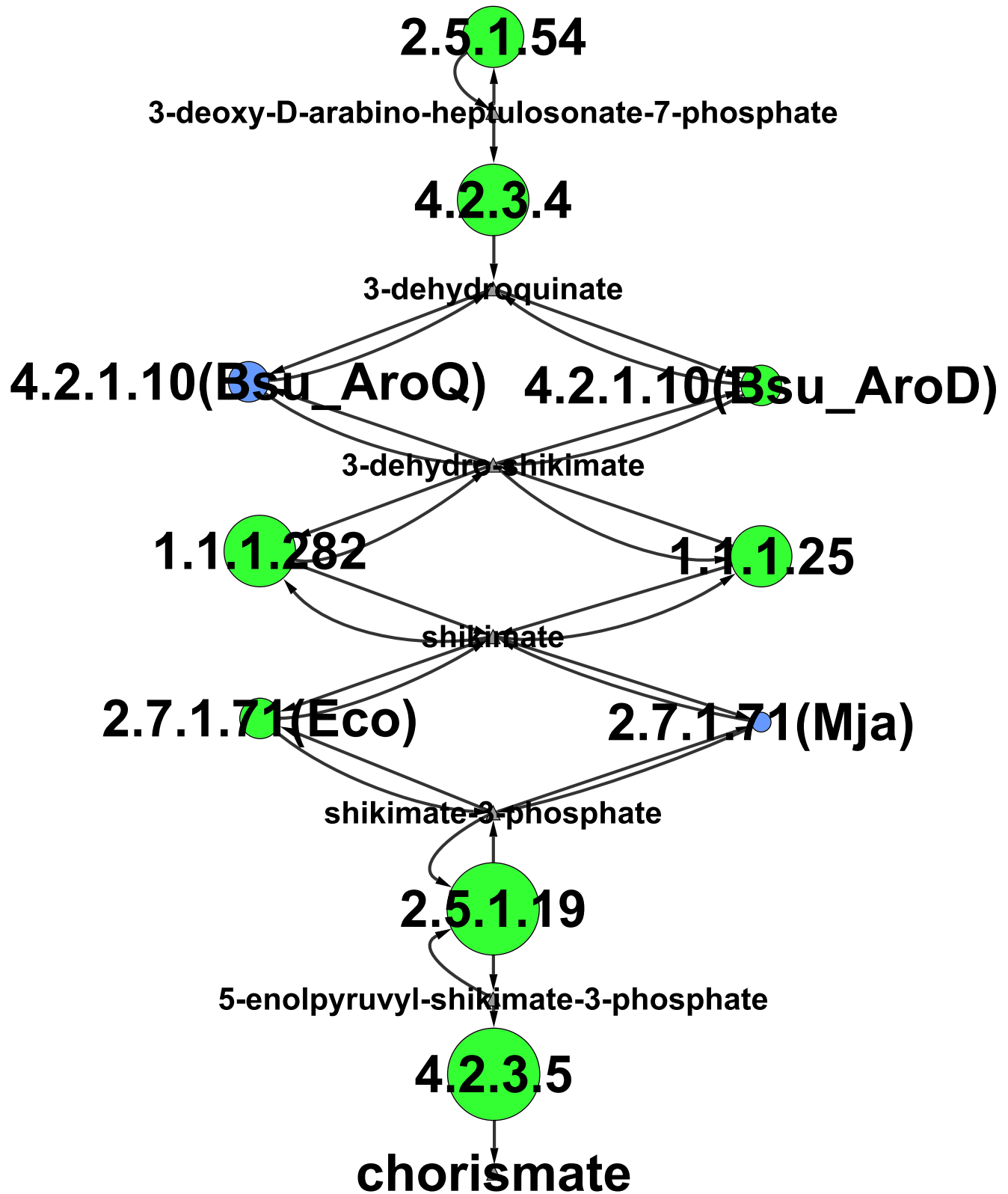

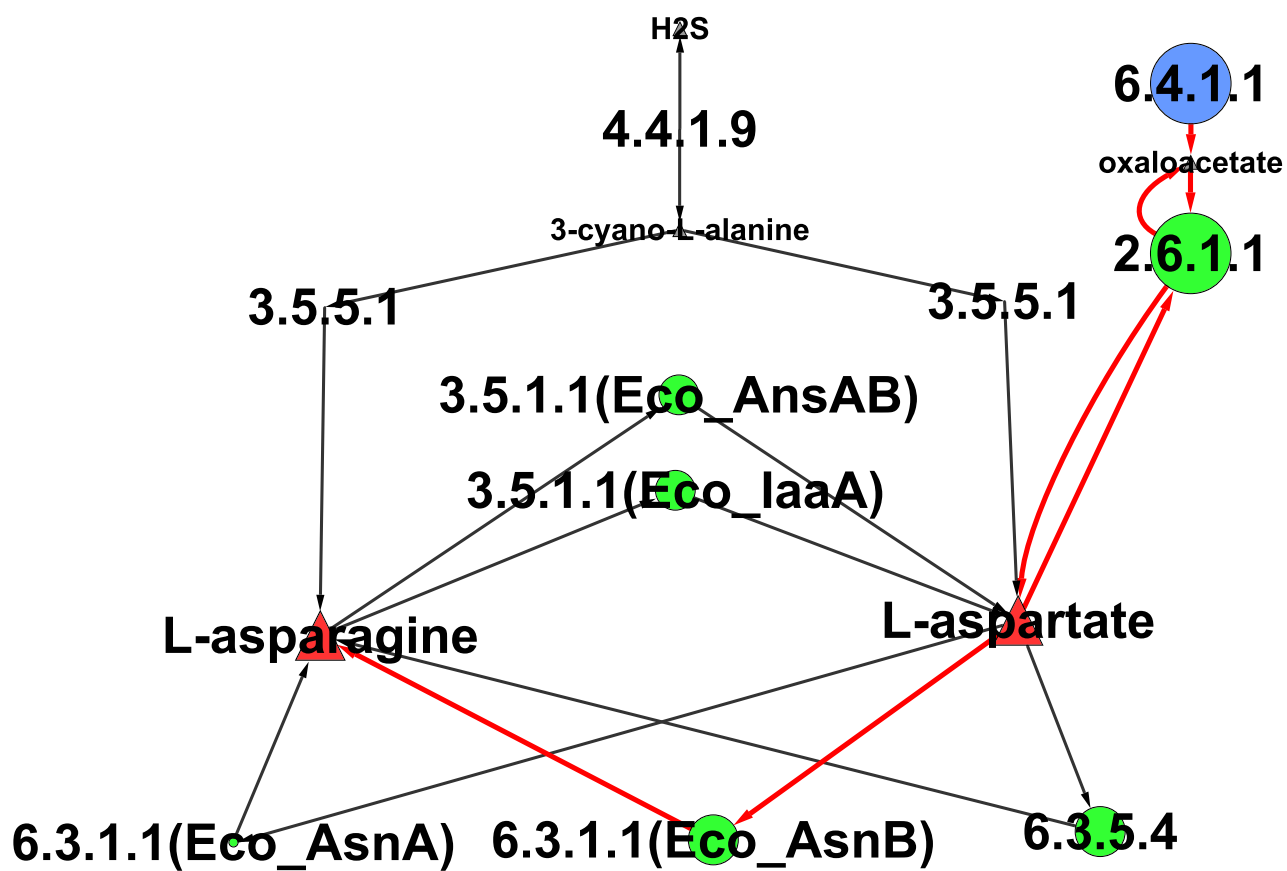

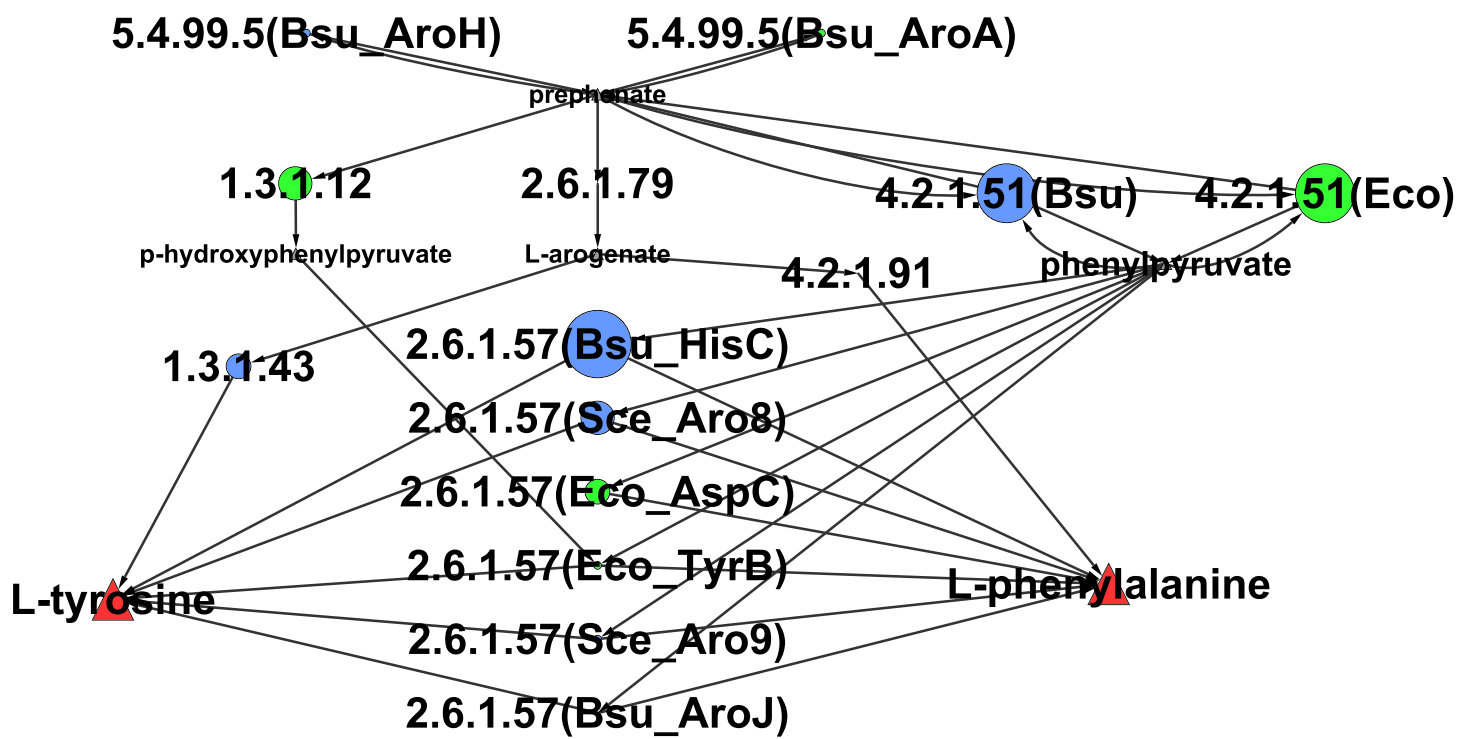

Supplement: Additional data file 3 — Detailed view from Figure 1 for pathways in the following order: (a) Arg, (b) Gly-Thr, (c) Trp, (d) Pro, (e) Ile-Val-Leu, (f) His, (g) Glu-Gln, (h) Ala-Cys-Ser-Met, (i) Lys, (j) Cor, (k) Asp/Asn, (l) Phe/Tyr. Nomenclature is as in Figure 1. [file gb-2008-9-6-r95-S3.pdf]
